# High-Throughput Genotyping of Common Chromosomal Inversions in the Afrotropical Malaria Mosquito *Anopheles Funestus*

**DOI:** 10.3390/insects11100693

**Published:** 2020-10-13

**Authors:** Martin Lukindu, R. Rebecca Love, Moussa W. Guelbeogo, Scott T. Small, Melissa T. Stephens, Nathan R. Campbell, N’Fale Sagnon, Carlo Costantini, Nora J. Besansky

**Affiliations:** 1 Eck Institute for Global Health, University of Notre Dame, Notre Dame, IN 46556, USA; mlukindu@nd.edu (M.L.); rlove1@nd.edu (R.R.L.); ssmall2@nd.edu (S.T.S.); 2 Department of Biological Sciences, University of Notre Dame, Notre Dame, IN 46556, USA; stephens.49@nd.edu; 3 Centre National de Recherche et Formation sur le Paludisme (CNRFP), Ouagadougou, Burkina Faso; guelbcnrfp@yahoo.fr (M.W.G.); sagnon_cnrfp@yahoo.fr (N.S.); carlo.costantini@ird.fr (C.C.); 4 GTseek LLC, Twin Falls, ID 83301, USA; Nathan.campbell@gtseek.com; 5 5 MIVEGEC, University of Montpellier, CNRS 5290, IRD 224, F-34394 Montpellier, France

**Keywords:** *Anopheles funestus*, chromosomal inversion polymorphism, polytene chromosome analysis, inversion genotyping, karyotyping, malaria vector, tag SNP

## Abstract

**Simple Summary:**

Chromosomal inversions occur when a segment of DNA breaks in two places, rotates 180 degrees, and reattaches. Inversions can protect sets of genetic variants, including those adapted to local conditions, from being split up in the random shuffling that occurs when genetic information is transmitted from one generation to the next. For this reason, inversions can play a role in local adaptation and range expansion. Like many malaria mosquitoes, *Anopheles funestus*, which plays a major role in transmitting malaria in sub-Saharan Africa, carries several common chromosomal inversions. Three of these inversions have been implicated in traits such as indoor resting behavior, which affects their rate of contact with both humans and insecticide-based interventions. Inversions therefore have relevance for malaria epidemiology and control. Inversions are traditionally identified by examining chromosomes under a microscope, but this method is difficult and time-consuming, and only applicable to a subset of female adult mosquitoes. To overcome this limitation, we developed high-throughput DNA-based diagnostic methods to predict the presence of inversions. The availability of these methods will allow scientists to more easily identify inversions in larger samples of mosquitoes, from all life stages and both sexes, which will help us determine how inversions are affecting malaria transmission.

**Abstract:**

Polymorphic chromosomal inversions have been implicated in local adaptation. In anopheline mosquitoes, inversions also contribute to epidemiologically relevant phenotypes such as resting behavior. Progress in understanding these phenotypes and their mechanistic basis has been hindered because the only available method for inversion genotyping relies on traditional cytogenetic karyotyping, a rate-limiting and technically difficult approach that is possible only for the fraction of the adult female population at the correct gonotrophic stage. Here, we focus on an understudied malaria vector of major importance in sub-Saharan Africa, *Anopheles funestus*. We ascertain and validate tag single nucleotide polymorphisms (SNPs) using high throughput molecular assays that allow rapid inversion genotyping of the three most common *An. funestus* inversions at scale, overcoming the cytogenetic karyotyping barrier. These same inversions are the only available markers for distinguishing two *An. funestus* ecotypes that differ in indoor resting behavior, Folonzo and Kiribina. Our new inversion genotyping tools will facilitate studies of ecotypic differentiation in *An. funestus* and provide a means to improve our understanding of the roles of Folonzo and Kiribina in malaria transmission.

## 1. Introduction

Paracentric chromosomal inversions result from the breakage and end-to-end reversal of a segment of one chromosome arm. This type of chromosomal rearrangement is ubiquitous across plant and animal species [1,2,3], but it has been most closely studied in dipterans—notably *Drosophila*, *Simulium*, and *Anopheles* mosquitoes—whose giant polytene chromosomes [4] form distinct banding patterns that allow paracentric inversions to be readily observed through microscopy. Closely related species in these groups are often distinguished by one or more fixed inversion differences, and intraspecific inversion polymorphism is common [5,6,7]. Theory suggests that the evolutionary significance of inversions stems from suppressed recombination in the rearranged region of chromosomal inversion heterozygotes [3,8]. If an inversion captures locally adapted allelic combinations, it can maintain them as haplotype blocks protected from homogenization with other genetic backgrounds.

Although lacking a basis in formal genetic modeling, Coluzzi’s 1982 theory of ecotypification [9] proposed a role for chromosomal inversions in ecological differentiation and speciation of anophelines (and other dipteran groups) well in advance of the quantitative and empirical support for this role that exists today [1,2,3,8]. In his “verbal” model, Coluzzi [9] noted that dipteran groups like *Drosophila*, *Simulium*, and *Anopheles* share characteristics that might make them prime candidates for paracentric inversion-influenced speciation, among them low chromosome number, active dispersal, and high vagility relative to environmental heterogeneities that can be perceived by these insects. The observations underpinning his model came from arguably the most extreme dipteran group in this regard, *Anopheles* mosquitoes, with high dispersal ability and only three chromosomes. His model envisioned a species such as *Anopheles gambiae* distributed across a spatially heterogeneous environment, with recurring “boom-bust” demographic cycles associated with temporal (dry/wet seasonal) heterogeneities. Inversions arising in populations locally adapted to conditions at the ecological or geographic margins of the species range would capture allelic variation that could facilitate range expansion, while also permitting more efficient utilization of spatial and temporal heterogeneities in the central range.

Among anophelines, paracentric chromosomal inversions have been most extensively studied in the Afrotropical sibling species group known as the *Anopheles gambiae* complex, in which at least 31 fixed and polymorphic inversions have been described [7,10]. The most chromosomally polymorphic and geographically widespread members of this group are also major vectors of human malaria throughout sub-Saharan Africa, a disease that claims over 435,000 lives in this region each year [11]. These mosquito vectors show clinal variation in inversion frequencies that follow altitudinal and latitudinal climatic gradients [7,12]. The same inversions associated with arid geographic regions are also more frequent in arid microclimates and in the dry season at local spatial scales [7,13]. Field and laboratory studies suggest that these inversions confer ecologically relevant phenotypes (e.g., thermal and desiccation resistance) and that they are the targets of strong balancing (spatially varying) selection [14,15,16,17,18,19,20]. The implication that inversions influence epidemiologically relevant anopheline behavior and physiology, such as adult indoor resting and biting, host choice, and *Plasmodium* susceptibility [21,22,23], adds public health significance to the fundamental but largely unsolved problems of defining more precisely the physiological and behavioral phenotypes influenced by inversions, and detailing their underlying molecular basis.

Broadly co-occurring throughout sub-Saharan Africa with the primary vectors in the *An. gambiae* complex, *Anopheles funestus* is another primary African malaria vector of equal or sometimes greater efficiency, but one that remains relatively understudied [24,25,26]. Importantly, *An. funestus* extends malaria transmission into the dry season, peaking in abundance at this time after the populations of other vector species have waned. Cytogenetic studies have revealed at least 17 paracentric chromosomal inversions segregating in *An. funestus* [27,28,29,30,31]. Spatially explicit modeling of seven of these rearrangements at a macroecological level in West-Central Africa showed that the frequencies of most of the studied inversions were significantly correlated with environmental gradients of precipitation, similar to patterns in the *An. gambiae* complex [12]. Investigations in the Central African country of Cameroon implicated *An. funestus* inversions in local adaptation and assortative mating [32,33], as well as wing shape variation [34]. In the West African country of Burkina Faso, strictly sympatric but assortatively mating ecotypes of *An. funestus*, referred to as Folonzo and Kiribina chromosomal forms, are marked by contrasting degrees of inversion polymorphism [35,36]. They differ both in seasonal abundance and in epidemiologically relevant indoor resting behavior [37,38], but are morphologically indistinguishable, and the only method of identification to-date relies on karyotyping [36]. Further study of any of these phenomena in *An. funestus* requires cytogenetic analysis.

Traditional cytogenetic karyotyping relies on observation of banding patterns in polytene chromosomes under a phase-contrast microscope. This approach is restrictive for any anopheline species, as the most favorable polytene chromosomes (those for which cytogenetic maps are typically designed) develop in the ovarian nurse cells after blood feeding. Accordingly, cytogenetic karyotyping is limited to adult females at a specific gonotrophic stage, and only those whose chromosomes have been adequately fixed in the appropriate solution and preserved at the correct (cold) temperature [39]. Furthermore, this approach requires experienced cytogeneticists and is time consuming, and hence difficult to scale up. For *An. funestus* in particular, these difficulties are compounded because its polytene chromosomes are relatively difficult to work with [36]. Recently, we have developed approaches for molecular inversion genotyping based on tag single nucleotide polymorphisms (tag SNPs) that are highly correlated with inversion orientation in *An. gambiae* and *An. coluzzii* in the *An. gambiae* complex [40]. Tag SNPs were computationally ascertained in the genomic database of natural variation for these species (Ag1000G; [41]), and high throughput assays to genotype candidate tags in individual mosquitoes were developed based on probe hybridization and amplicon sequencing [42]. We found that the two molecular methods performed comparably when applied to DNA from the same set of previously cytogenetically karyotyped mosquitoes, and they rivaled traditional cytogenetic karyotyping not only in genotyping speed but also in accuracy.

Here, we implement a strategy similar to that used in *An. gambiae s.l*. to achieve high throughput genotyping of three of the most highly polymorphic and geographically widespread inversions in *An. funestus*, notably those used to discriminate the Folonzo and Kiribina ecotypes (3Ra, 3Rb, 2Ra). In the absence of a database comparable to Ag1000G for *An. funestus*, we performed deep Illumina sequencing of 186 previously cytogenetically karyotyped *An. funestus* from Burkina Faso, where Folonzo and Kiribina co-occur, and 15 unkaryotyped *An. funestus* from six other countries. After calling variants in the rearranged regions with respect to the recent chromosome-based *An. funestus* assembly [43], we computationally ascertained candidate tag SNPs and developed high throughput hybridization and amplicon sequencing assays to genotype the tags. We validated the candidate tags using DNA extracted from a second collection of cytogenetically karyotyped *An. funestus* from Burkina Faso. We achieved relatively high concordances between molecularly and cytogenetically assigned karyotypes for each of the targeted inversions in *An. funestus*. Our study provides tools to advance the study of inversion-influenced ecotypification in this important but neglected vector species.

## 2. Materials and Methods

### 2.1. Illumina Whole Genome Sequencing and Ascertainment of Candidate Tag SNPs

#### 2.1.1. Mosquito Sampling and Sequencing

Adult *An. funestus* samples used for whole genome sequencing and candidate tag SNP ascertainment were from historical collections, conducted indoors by pyrethrum spray catch from 11 villages in the Sudan-Savanna or Guinean-Savanna ecoclimatic zones of Burkina Faso, between 2000 and 2002 (Supplementary Table S1). Ovaries from females at the appropriate gonotrophic stage had been dissected in the field and placed into Carnoy’s solution in individual tubes labeled with unique serial numbers (one per mosquito) for subsequent cytogenetic karyotyping. The corresponding carcass was preserved in a separate tube with desiccant and assigned the same mosquito-specific serial number. Detailed description of the study area, methods of field sampling and processing, molecular identification, and polytene chromosome analysis are provided in Costantini et al. [35] and Michel et al. [44].

To mitigate against geographic bias, we obtained *An. funestus* samples previously field-collected between 2001 and 2014 from six additional countries in West, East, and Southern Africa (Ghana, Kenya, Tanzania, Uganda, Mozambique, Zambia) (Supplementary Table S1). These had been identified molecularly [45,46] and preserved individually as desiccated adults, but none had been cytologically karyotyped.

Genomic DNA was individually extracted from a combined total of 202 specimens from seven African countries (Supplementary Table S1), following a CTAB protocol [47]. Shotgun DNA library preparation and Illumina sequencing were performed at McGill University and Génome Québec Innovation Center (Montreal, Canada) using the NEBNext Ultra II DNA Library Prep Kit (New England Biolabs, Ipswich, MA, USA) and the HiSeq X with 150 paired-end cycles. Adapter sequences and low-quality bases were removed from sequencing reads using trim_galore (github.com/FelixKrueger/TrimGalore). Read pairs with one read shorter than 75 base pairs were removed. Trimmed reads were decontaminated by aligning to a custom file of bacteria (*Pantoea sp., Asaia bogorensis*, *Enterobacter asburiae*, *Klebsiella oxytoca*, *K. variicola*, and *Pseudomonas aeruginosa*) and PhiX genomes using BWA v.0.7.15 [48], with only unmapped read pairs retained. Processed reads (trimmed and decontaminated) were then aligned to the *An. funestus* AfunF3 reference assembly [43] using BWA.

Variants were called separately for each individual mosquito using GATK v.3.5 [49] and HaplotypeCaller with options: -emit-ref-confidence GVCF -heterozygosity 0.01 -indel-heterozygosity 0.001 -min-base-quality-score 17. Variant filtering was done in two steps. First, the resulting GVCFs produced by HaplotypeCaller were genotyped using GenotypeGVCFs. Variants were filtered based on the following metrics: quality by depth (QD) < 5, quality (QUAL) < 30, depth (DP) < 14, mapping quality (MQ) < 30, MQRankSum < −12.5, ReadPosRankSum < −8.0, strand bias (FS) > 60.0. Filtered GVCFs were then merged into a single species GVCF using CombineGVCFs followed by GenotypeGVCFs. Second, genotypes with a GQ < 30 and DP < 20 were marked as missing. Variant quality was evaluated using scikit-allel v1.1.0 (doi:10.5281/zenodo.2652508) following Reference [50]. Sites were masked as repetitive using RepeatMasker [51] with a custom repeat file [52]. Sites were also masked if they had read coverage outside of the bounds defined by +/− 3*sqrt(avgCov per chromosome) or were identified as paralogs using the methods outlined in SNPable (http://lh3lh3.users.sourceforge.net/snpable.shtml).

#### 2.1.2. Tag SNP Discovery

Although cytogenetic karyotype information was available for the mosquito sample from Burkina Faso, this information was lacking for the specimens collected from other countries. Fortunately, independent of knowledge (or lack thereof) about cytogenetic karyotypes, it is possible to infer inversion genotypes from population-based high-density SNP genotype data, owing to the population substructure created by suppressed recombination in the inverted region. Ma and Amos [53] demonstrated that the application of principal components analysis (PCA) to SNP genotypes specifically within the local window of the genome containing an inverted region produces a pattern indicative of two distinct “populations” of inversion homozygotes (inverted and standard) and their 1:1 admixture (inversion heterozygotes). In a plot of the first two principal components, this manifests as three equidistant stripes, where the outer stripes represent alternative homokaryotypes, and the middle stripe represents the inversion heterokaryotype [53]. This information alone allows the population sample to be classified as homozygous or heterozygous, but it cannot determine the inversion orientation of the two homozygous outer stripes. However, if the population subject to this type of local PCA contains a subset of specimens with known cytogenetic karyotypes, the genotype of the outer stripes can be inferred based on their inclusion within an outer stripe. In principle, this method allows individuals in an entire population sample to be genotyped for an inversion, assuming that the inversion is non-recurrent evolutionarily, and that other sources of population structure (e.g., geographic) that could obscure a clear three-stripe pattern are minimal. PCA-based inversion genotyping can be definitive if the assumptions are met, but the PCA must be performed on population genomic data—not on individual sequences—and is successful only for those populations sufficiently polymorphic to allow the expected three-stripe pattern. Our goal was to identify candidate tag SNPs predictive of inversion genotypes in individual mosquitoes.

As a first step toward that goal, we exploited the local PCA approach just described to impute inversion genotypes at 2Ra, 3Ra, and 3Rb computationally. We used the high-density population genomic SNP data of specimens from seven African countries (Supplementary Table S1), limiting consideration to those variants predicted to fall within the breakpoints of each inversion. The precise inversion breakpoint locations are not known, as they have not been characterized molecularly. Accordingly, genomic coordinates corresponding to estimated breakpoint locations (Table 1) were inferred based on DNA markers physically mapped to the polytene chromosomes, with reference to the *An. funestus* cytogenetic photomap [31]. Following an approach similar to that described for *An. gambiae* and *An. coluzzii* [40], we considered only biallelic SNPs whose minor allele count was ≥4. For each inversion (2Ra, 3Ra, 3Rb) and mosquito, we created a matrix of one-digit genotypes at these SNPs, by converting the biallelic SNP genotype into a count of alternate alleles (where an alternate allele is one that does not match the AfunF3 reference at the focal position). Thus, SNP genotypes at candidate tags were coded as 0, 1, or 2 if zero, one, or two alternate alleles were present at that position. Following Reference [53], we computationally imputed inversion genotypes using PCA of the SNP genotype matrix, with functions in scikit-allel [50]. We represented the output as a scatter plot of the first two principal components for each mosquito in the population sample. The correct genotype corresponding to the two homokaryotype stripes was determined based on the inclusion in a given stripe of mosquitoes with cytologically determined karyotypes. Based on this classification, mosquitoes without cytologically determined karyotypes could be assigned a PCA karyotype.

This approach depends upon the observation of a three-stripe pattern in plots of PC1 and PC2. Specimens not conforming to this expected pattern (28 of 201), presumably due to cryptic population structure or admixture with an unsampled population, could not be confidently genotyped by PCA and were thus excluded from tag SNP development (Supplementary Table S1, Figure S1), although for the benefit of future research, we list the excluded specimens on a separate page of Supplementary Table S1. Of the remaining 173 specimens whose genotype was imputed by PCA, 158 from Burkina Faso also had been cytogenetically karyotyped. Mismatches between cytogenetic and PCA assignments were detected at a level of 6.3% for inversion 2Ra, 4.4% for 3Ra, and 6.5% for 3Rb. Based on the PCA plots (Supplementary Figure S1), and analogous PCA-based karyotyping results for *An. gambiae* and *An. coluzzii* specimens with previous cytogenetic karyotype assignments [40], these mismatches are most reasonably interpreted in terms of errors arising from cytogenetics (inference or recording). Using this PCA-based inversion genotyping approach, we were able to impute inversion genotypes for the geographically dispersed specimens that had not been cytogenetically karyotyped (Supplementary Figure S1, Table S1).

In the last step, we calculated the concordance between the PCA-based inversion genotype (0 = standard homozygote, 1 = inversion heterozygote, 2 = inversion homozygote) and each candidate tag SNP genotype (0 = homozygous reference allele, 1 = heterozygote, 2 = homozygous alternate allele) for all mosquitoes in the sample that could be genotyped at the focal SNP and by PCA. SNPs capable of accurately predicting inversion genotype should have allelic states that are strongly correlated with inversion status. Given that the Fumoz reference represents an un-inverted karyotype across the genome, reference alleles at candidate tags are expected to be associated with standard arrangements, while alternate alleles should be associated with inverted arrangements. For each candidate tag SNP in a focal inversion (2Ra, 3Ra or 3Rb), we measured the proportion of mosquitoes in the sample whose PCA-based inversion genotype, expressed as the number of chromosomes carrying the inversion in question, matched the SNP genotype, expressed as the number of alternate alleles. Candidate tags were defined as those whose genotypes agreed with the PCA-based genotype in at least 80% of mosquitoes.

Our ultimate goal was to ascertain biallelic SNPs whose alternative alleles were strongly (>80%) correlated with opposite orientations of the inversions of interest (2Ra, 3Ra, 3Rb). The association between alleles at a SNP and inversion orientation in a population polymorphic for the inversion is maintained by suppressed recombination inside a heterozygous inversion region and potentially also by selection, but the association is typically not absolute because of double crossover events and gene conversion. Importantly, no individual candidate tag SNP need be perfectly deterministic of inversion orientation for successful genotyping, because the intended high throughput molecular genotyping approaches (Section 2.3 and Section 2.4, below) accommodate the scoring of tens or hundreds of SNPs. When scored in aggregate, several strongly but imperfectly correlated SNPs should accurately reflect inversion orientation.

### 2.2. Mosquito Samples for Validation of Tag SNPs

A second historical *An. funestus* collection was used for validation of candidate tags (Supplementary Table S2). With one exception, mosquitoes used for validation of tag SNPs were sampled from two rural villages located ~2 km apart in the Sudan savanna vegetation belt, Koubri (12°11′54 N; 1°23′43 W) and Kuiti (12°11′36 N; 1°23′11 W). An additional mosquito was collected from a nearby rural village, Noungou (12°32′ N; 1°24′ W). As the collections were performed in the framework of previous longitudinal studies of *An. funestus* behavior, multiple methods of adult sampling were employed (resting catch indoors and from outdoor pit shelters, insecticide spray catch indoors, human landing catch indoors and outdoors, and odor-baited entry trap; Supplementary Table S2). Mosquitoes and ovaries were processed as described in Section 2.1.

### 2.3. TaqMan OpenArray Assay Design and Workflow for Genotyping of Inversions 3Ra and 3Rb

TaqMan OpenArray (OA) SNP genotyping assays were designed at an early stage in the assembly of the chromosome-based AfunF3 reference genome [43], thus for logistical reasons, we focused exclusively on inversions 3Ra and 3Rb. Beginning with the list of candidate tag SNPs ascertained as described in Section 2.1, we filtered out any candidate tag whose flanking sequence was unsuitable for the design of forward and reverse PCR primers (e.g., rich in low complexity or repetitive DNA), as well as any candidate containing other polymorphic sites within 30 bases of the tag SNP, as judged from our whole genome variation data (Section 2.1). Designs for the remaining 28 tag SNPs (16 in 3Ra and 12 in 3Rb) were produced by the Dana-Farber/Harvard Cancer Center (DF/HCC) Genotyping and Genetics for Population Sciences Core, a unit of the Partners HealthCare Center for Personalized Genetic Medicine. Each assay consists of forward and reverse PCR primers which produce ~100 base amplicons containing the tag SNP, and two allele-specific VIC- or FAM-labeled ‘reporter’ probes to discriminate between the reference and alternate alleles at the tag. Primers and probes for each of the 27 assays that ultimately passed quality control (15 3Ra and 12 3Rb assays; see Section 2.5) are provided in Supplementary Table S3. Based on the initial 28 tags, we selected a custom 32-array genotyping plate design (Thermo Fisher Scientific, Waltham, MA, USA) that genotypes 96 mosquitoes at 28 tags (2688 genotypic assays) per plate.

Quantification of genomic DNA extracted from the validation specimens (Section 2.2) was conducted by DF/HCC via picogreen-based fluorimetry, and average DNA concentration was 7.5 ng/uL (standard deviation [SD] = 3.6). Because OA requires only 250 copies of a haploid genome for each individual through-hole (0.0675 ng of *An. gambiae* genomic DNA, assuming a haploid genome size of 0.27 pg; [54]), 28 through-holes per mosquito require a total of only ~2 ng DNA. DF/HCC performed the genotyping using endpoint detection of fluorescent signals on the TaqMan OpenArray Genotyping System, following manufacturer’s specifications (Applied Biosystems, Foster City, CA, USA). Conditions for genotyping are available upon request to DF/HCC.

### 2.4. Amplicon Sequencing Assay Design and Workflow for Genotyping of Inversions 2Ra, 3Ra, and 3Rb

We used the multiplexed amplicon sequencing (AS) approach called GT-seq (Genotyping-in-Thousands by sequencing; [55]). As a first step, candidate tag SNPs were evaluated with respect to primer design and primer pooling using custom perl scripts [55], resulting in 29, 33, and 28 assays designed for tag SNPs in 2Ra, 3Ra, and 3Rb, respectively. Following Reference [55], Illumina sequencing primer sites were added to locus-specific forward and reverse primer sequences to create PCR1 primers, which were ordered along with PCR2 primers (a set of 96 i5 and i7 indexes) from Integrated DNA technologies in 96-well plate format at a 25 nmole synthesis scale and a concentration of 200 µM in Tris-EDTA pH 8.0 buffer (TE). GT-seq test libraries were prepared and sequenced by the University of Notre Dame Genomics and Bioinformatics Core Facility (GBCF) to identify primers that produced polymerase chain reaction (PCR) artefacts or were overrepresented. Following optimization, primer pools were re-made to include only the optimized panel of PCR1 primers. Tag SNPs and PCR1 primer pairs for GT-seq genotyping are listed in Supplementary Table S4 for the 25 2Ra, 31 3Ra, and 26 3Rb tags retained following quality control.

The final libraries prepared by the GBCF were constructed without optional exo-SAP treatment following Reference [55] except for the following modifications to PCR conditions and post library cleanup: PCR1: 95 °C—15 min; 5 cycles (95 °C—30 s, 3% ramp down to 57 °C—30 s, 72 °C—2 min); 10 cycles (95 °C—30 s, 65 °C—30 s, 72 °C—30 s); 4 °C hold. PCR2: 95 °C—15 min; 10 cycles (95 °C—10 s; 62 °C—30 s; 72 °C—30 s); 72 °C—5 min; 4 °C hold. At the conclusion of PCR2, each plate of samples was purified and normalized using the Just-a-Plate 96 PCR Purification and Normalization Kit (Charm Biotech) according to the manufacturer’s instructions. After normalization, 10 uL of each sample per 96-well plate (up to 960 uL total) was then combined into a 1.5 mL Eppendorf tube, for a total of 10 tubes. From each tube, 300 uL was transferred to a fresh 1.5 mL Eppendorf tube for two rounds of purification using AMPure XP paramagnetic beads (Beckman Coulter Life Sciences, Indianapolis, IN, USA) with ratios of 0.5X and 1.3X, respectively. Purified libraries were eluted in 35 uL 1 x TE and transferred to fresh 1.5 mL tubes before adding 3.5 uL buffer EB containing a 1% Tween 20 solution.

Each of the libraries was quality assessed on an Agilent Bioanalyzer 2100 High Sensitivity chip and quantified by quantitative PCR (qPCR) using the Illumina Kapa Library Quantification Kit (Roche, Cat. #KK4824). The libraries were then normalized to a concentration of 4 nM and pooled for sequencing. The final pooled library containing 235 *An. funestus* individuals (as well as another pooled library containing 957 *An. gambiae* and *An. coluzzii* individuals from a separate study [42]) was sequenced on a single lane of Illumina NextSeq 500 v2.5 (75 cycle) High Output flowcell using a dual indexed 75 bp single-end read. Base calling was done by Illumina Real Time Analysis (RTA) v2 software.

Using scripts described in the bioinformatics pipeline of Reference [55] and available on Github (https://github.com/GTseq), sequencing data were demultiplexed into single fastq files for each individual sample. Individuals were genotyped at each locus with a perl script (GTseq_Genotyper_v3.pl) that counts the occurrence of each allele at a locus within individual fastq files. The ratio of allele 1 to allele 2 counts was used to generate a genotype for each locus with total read counts > 10, following the methods and cut-offs of Reference [55].

### 2.5. Converting Genotypes at Individual Tags to Multilocus Inversion Genotypes

For both OA and AS approaches, data quality was checked using two measures. First, we assessed the tag SNP call rate, a tag-specific value representing the percentage of mosquito specimens in the sample with a genotype call at the focal tag. Any tag with a call rate < 80% was eliminated from the genotyping panel. Second, we calculated the specimen call rate, a specimen-specific value representing the percentage of tag SNPs that could be confidently genotyped in a focal mosquito. If the specimen call rate was <80%, that mosquito specimen was excluded from further analysis.

Following data quality filtering, a multi-locus inversion genotype was calculated for each mosquito based on a custom python script. Genotypes at individual tag SNPs represent the count of alternate alleles at that tag. The multi-locus inversion genotype represents the average number of alternate alleles across all tag SNPs scored in a given inversion. Binning of this average (0–0.67, 0.68–1.33, 1.34–2) produces the predicted inversion genotype of 0, 1, and 2, respectively.

### 2.6. Code and Data Availability

Data can be found at https://figshare.com/projects/Anopheles_gambiae_An_coluzzii_and_An_funestus_molecular_inversion_karyotyping_raw_amplicon_sequencing_data/81128 and https://figshare.com/projects/Anopheles_funestus_SNPs_within_inverted_regions/89024, and in the Sequence Read Archive under BioProject ID PRJNA660016, accession numbers SAMN15932485 to SAMN15932719. Code used to generate the data can be found on Github (https://github.com/GTseq and https://github.com/rrlove/molec_karyo_notebooks).

## 3. Results

Candidate tag SNPs whose allelic state was strongly correlated with chromosomal inversion genotype were ascertained based on whole genome sequences of cytogenetically karyotyped *An. funestus* from Burkina Faso (Methods, Section 2.1). Tag SNP genotyping assays reliant on probe hybridization (TaqMan OpenArray, henceforth OA) or amplicon sequencing (GT-seq, henceforth AS) were developed, and we performed one or both molecular genotyping approaches on an independent sample of cytogenetically karyotyped *An. funestus* from Burkina Faso for validation (Methods, Section 2.2, Section 2.3, Section 2.4 and Section 2.5).

### 3.1. OA Genotyping

Custom 32-array OA genotyping plates were used to genotype 238 individual *An. funestus* mosquitoes at 28 tag SNP loci. One of the 28 tags was not genotyped successfully in at least 80% of the sample (only 74.8% of specimens were called at this tag; Supplementary Figure S2), thus we eliminated it from the final tag SNP panel (Supplementary Table S3). After filtering, the remaining 27 tags had call rates ranging from 97.5% to 100% (mean, 99.7%; SD, 0.55). After removing low-quality tag SNPs, the 238 specimens had an average specimen call rate of 99.7% (SD, 1.62; range, 81.5–100%; Supplementary Figure S2).

### 3.2. AS Genotyping

Sequencing from one NextSeq lane included the pooled GT-seq library of 235 *An. funestus* mosquitoes (Supplementary Table S2), as well as another GT-seq pooled library of 957 *An. gambiae* and *An. coluzzii* mosquitoes pertaining to an independent experiment [42]. This produced ~359M total reads, of which ~74M could be assigned to the 235 *An. funestus* specimens based on their barcode sequences. Read counts per individual mosquito averaged 314,786 (SD 183,680). The tag SNP call rate was below the 80% threshold for eight tags (Supplementary Figure S2), which were subsequently dropped from the final genotyping panel (Supplementary Table S4). After filtering, SNP call rates at the remaining 82 tags averaged 95.5% (SD 4.23; range, 82.1% to 99.1%). Five mosquito specimens were dropped due to low specimen call rates (Supplementary Figure S2). Of the remaining 230, the average specimen call rate was 96.7% (SD 3.67; range, 84.1% to 100%).

### 3.3. Concordance among Cytogenetic and Molecular Inversion Genotyping Methods

**Figure 1:**
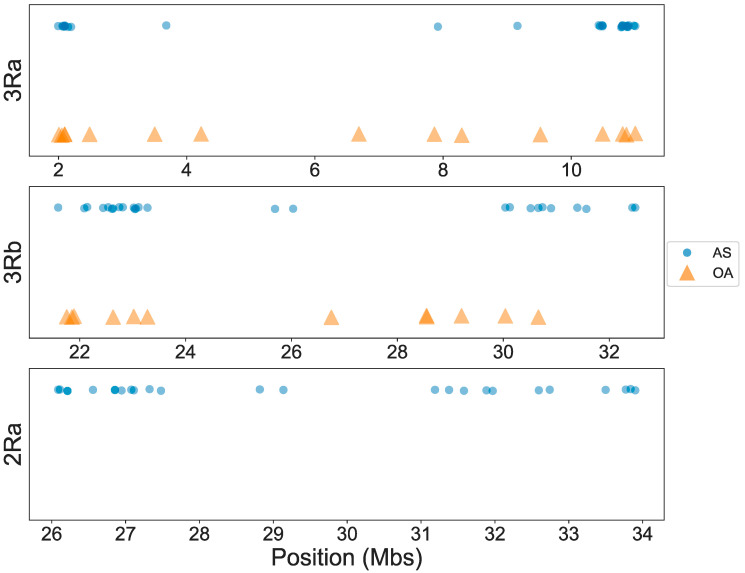
Locations of tag single nucleotide polymorphisms (SNPs) within each inversion for OpenArray (OA, orange triangles) and amplicon sequencing (AS, blue circles).

**Figure 2:**
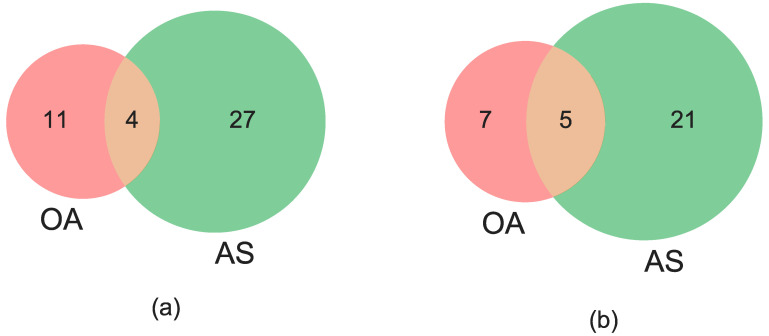
Venn diagrams showing degree of overlap between tag SNPs developed for Open Array (OA) and amplicon sequencing (AS) inversion genotyping of 3Ra (panel (**a**)) and 3Rb (panel (**b**)).

The approximate genomic positions of the final panel of OA and AS tag SNPs within inversions are shown in Figure 1. Although there appears to be an overrepresentation near inversion breakpoints, tags are distributed across most of the length of the inversions. Importantly, the precise genomic positions of the tag SNPs in the OA versus the AS genotyping panels for 3Ra and 3Rb are almost completely non-overlapping (Figure 2, Supplementary Tables S3 and S4), an outcome pursuant to the distinct filtering criteria for molecular assay development between the two methods. Although the tags within a given inversion are not statistically independent owing to tight linkage inside a chromosomal inversion, agreement between the OA and AS approaches based on different subsets of tags provides stronger evidence for inversion genotype inference than results based on one molecular method alone.

**Figure 3:**
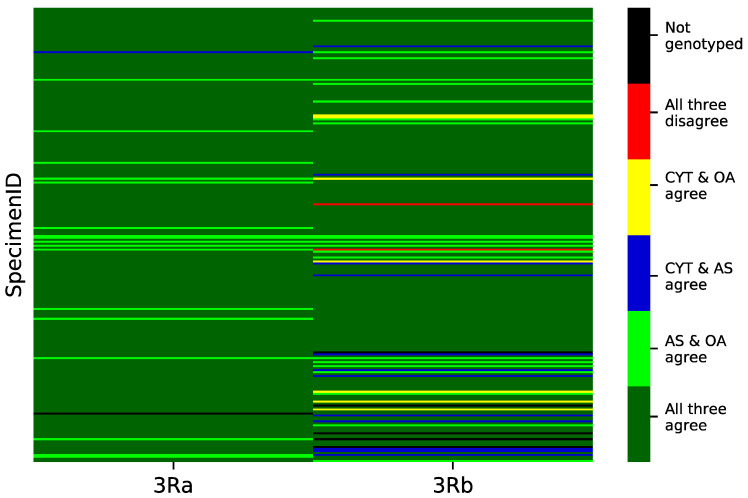
Concordance heat map of genotypes imputed by cytogenetics (CYT), OpenArray (OA), and amplicon sequencing (AS). Each row is an individual mosquito, and the columns (inversions 3Ra and 3Rb) report the degree of agreement among genotyping approaches for the inversions in each mosquito. Dark green represents 3-way genotypic concordance; light green, concordance between AS and OA; blue, concordance between CYT and AS; yellow, concordance between CYT and OA; red, 3-way discordance; black, missing data for at least one genotyping approach.

Table 2 and Figure 3 present genotypic concordance for the subset of specimens that were successfully scored by classical cytogenetics at a focal inversion. The numbers in Table 2 are based on the comparison between the cytogenetic genotype of a given specimen and the genotypes imputed by one (2Ra) or both (3Ra, 3Rb) molecular approaches from the same specimen (concordance with PCA genotypes is treated in the Discussion Section). For 3Ra, of the 229 mosquitoes successfully scored by all three methods, 211 (92%) had fully concordant genotypes. Of the 18 specimens with discordant genotypes, 17 had cytogenetic genotypes that disagreed with concordant molecular genotypes, consistent with the possibility of cytogenetic karyotyping error. For 3Rb, 225 mosquitoes were successfully scored by all three methods, which fully agreed for 183 (81%). There were 42 mosquitoes whose genotypes disagreed, 21 of which had cytogenetic genotypes conflicting with concordant molecular genotypes. Inversion 2Ra was scored only by cytogenetics and AS. Of 226 mosquitoes scored by both methods, 195 genotypes (86%) were fully concordant.

## 4. Discussion

If the cytogenetic genotype is considered a gold standard and taken as definitive, performance of the molecular assays was disappointingly low. However, several lines of evidence support the fact that classical cytogenetics is not infallible. World authorities in anopheline cytogenetics at the University of Rome La Sapienza periodically conducted double-blind investigations of their cytogenetic genotyping over the course of two decades, to assess the consistency of genotyping calls from the same set of slides. Error estimates ranged from 0% to 5% depending on slide quality [56], and could plausibly be higher for less experienced cytogeneticists. Evidence from a study of molecular inversion genotyping in *An. gambiae* in which cytogenetic karyotyping effort was randomly divided between two research groups was also consistent with a cytogenetic error rate as high as 5% [42]. Furthermore, a previous study of in silico inversion genotyping in *An. gambiae* compared cytogenetic genotypes and genotypes imputed from the corresponding sequenced mosquitoes by PCA using variation inside the inversion [40], similar to the procedure employed in the present study. Table 3 from that *An. gambiae* study [40] suggests an overall cytogenetic error rate of 4% (excluding one particular inversion with an anomalously high cytogenetic error rate). For the present study, neither the original chromosome preparation slides nor corresponding photomicrographs were available to confirm karyotype calls in cases of discrepancies. However, we can make a comparison between the cytogenetic genotype assignment and the PCA-based genotype inferred for the same specimen (Table 2, Supplementary Table S1), similar to what was done in the previous *An. gambiae* study [40]. For 2Ra, there are 9 mismatched assignments out of 143 mosquitoes, in which both cytogenetic and PCA assignments were available, pointing to a 6.3% cytogenetic error rate. Corresponding cytogenetic error rates for 3Ra and 3Rb were 4.4% and 6.5%, respectively. It is noteworthy that these rates are comparable to the frequencies of discordance in which the cytogenetic genotype differed from a common molecular (OA and AS) genotype, as the PCA genotypes were imputed from a different mosquito sample than the one employed for molecular inversion genotyping (Supplementary Table S1 versus Supplementary Table S2, respectively). Taken together, the evidence suggests that molecular inversion genotyping of *An. funestus* 2Ra, 3Ra, and 3Rb in Burkina Faso has an accuracy rate above 90%.

Consistent with the performance of OA and AS methods in *An. gambiae* [42], we found good concordance between both molecular genotyping approaches, suggesting that either one could be reasonably applied for inversion genotyping. The AS approach agreed slightly more often with cytogenetics than did OA (Table 2, Figure 3), and thus would be preferred, all else being equal. At least in part, the better performance of AS may be explained by the very high levels of nucleotide diversity found in *An. funestus* [57] that could interfere with the hybridization of two alternative 20-mer probes in the OA assays. The OA approach expects a perfect match between probe and chromosomal target, an expectation that depends on the study population segregating two haplotypes in the 20-base target region that differ only in the allelic state of the tag. Furthermore, as detailed by Campbell et al. [55], OA may be disadvantaged by higher genotyping costs relative to the AS approach (exemplified by GT-seq). Nevertheless, if the number of tag SNPs to be genotyped is low (50–100) and the number of samples is high (10^2^–10^3^), OA remains a cost effective option that is still widely used [55].

In contrast to the ascertainment of tag SNPs for inversion genotyping in *An. gambiae* [40], for *An. funestus,* we were constrained both by small sample size overall and limited numbers of specimens from geographic regions other than Burkina Faso. This may be one factor responsible for the apparently lower performance of molecular genotyping in *An. funestus* observed in this study relative to *An. gambiae* [42]. A public database of natural variation in *An. funestus* analogous to Ag1000G [41], broadly representative of the tropical African range of this species, is under development (M. Lawniczak, personal communication). Such a database will be necessary to verify or remedy the broad geographic applicability of the present tag SNP panels. Nevertheless, our focus on Burkina Faso was not accidental. The Folonzo and Kiribina chromosomal forms were discovered and have been most thoroughly studied in Burkina Faso [35,36,37,38,44,58], but the burden of cytogenetic karyotyping and absence of any other molecular taxonomic tool allowing their identification represented a severe impediment to further study of these epidemiologically relevant ecotypes. By analogy to the chromosomal forms MOPTI and SAVANNA in the *An. gambiae* complex, we anticipate that chromosomal inversions are instruments of ecotypic differentiation rather than taxonomic boundaries [59,60], but the cost-effective and high-throughput means of inversion genotyping developed here is a first step in improving our understanding of ongoing diversification within this major malaria vector.

## 5. Conclusions

To overcome the serious constraints of cytogenetic karyotyping of chromosomal inversions in *An. funestus*, we individually sequenced a collection of cytologically karyotyped mosquitoes and ascertained tag SNPs highly correlated with inversion genotype in inversions 2Ra, 3Ra, and 3Rb. We developed high throughput molecular assays that target these tags, both by probe hybridization and by amplicon sequencing, and validated these assays against an independent sample of cytologically karyotyped *An. funestus*. Both methods are more than 90% accurate, and because they can be performed at scale, they open up the possibility of studying the role of polymorphic inversions in the adaptive divergence of one of the most important malaria vectors in Africa.

## Figures and Tables

**Table 1:** Approximate genomic coordinates of inversion breakpoints.

Inversion	Genomic Coordinates
2Ra	25,967,767–33,984,223
3Ra	1,866,360–11,289,547
3Rb	20,512,400–33,000,000

**Table 2:** Concordance of genotypes imputed by cytogenetics (CYT), tag SNPs (AS, amplicon sequencing; OA, Open Array), and principal component analysis (PCA ^1^) for *An. funestus* inversions 3Ra, 3Rb, and 2Ra.

	3Ra	3Rb	2Ra
Concordance:			
CYT + AS + OA	211/229 (92.1%)	183/225 (81.3%)	NA
CYT + AS	---	---	195/226 (86.3%)
CYT + PCA	151/158 (95.6%)	145/155 (93.5%)	134/143 (93.7%)
Discordance:			
CYT vs. (AS + OA)	17/229 (7.4%)	21/225 (9.3%)	NA
(CYT + AS) vs. OA	1/229 (0.4%)	12/225 (5.3%)	NA
(CYT + OA) vs. AS	0/229 (0%)	7/225 (3.1%)	NA
CYT + AS + OA	0/229 (0%)	2/225 (0.9%)	NA
CYT vs. PCA	7/158 (4.4%)	10/155 (6.5%)	9/143 (6.3%)

^1^ PCA performed on genomic sequences of mosquitoes derived from a sample (Supplementary Table S1) independent of the one used for validation (Supplementary Table S2). NA, not applicable.

## Supplementary Materials

The following are available online at https://www.mdpi.com/2075-4450/11/10/693/s1, Figure S1: PCA-based genotyping of *An. funestus* inversions 2Ra, 3Ra, and 3Rb based on SNPs inside the rearranged regions, Figure S2: Tag SNP and specimen call rates for OpenArray and amplicon sequencing approaches, Table S1: Specimens used in tag SNP ascertainment with their cytogenetic and PCA-based inversion genotype assignments, Table S2: Specimens used in tag SNP validation with their cytogenetic and molecular (OA and AS) genotype assignments, Table S3: OpenArray tag SNP genotyping panel, Table S4: Amplicon sequencing tag SNP genotyping panel.
