# Supplementary material for: High-Throughput Genotyping of Common Chromosomal Inversions in the Afrotropical Malaria Mosquito Anopheles Funestus
Source: Insects. 2020 Oct 13;11(10):693. doi: 10.3390/insects11100693 (PMC7650614; doi:10.3390/insects11100693)
Supplement: Supplementary file 1 [file insects-11-00693-s001.zip › FigS1_PCA_with_legend_29Sep2020.pdf]

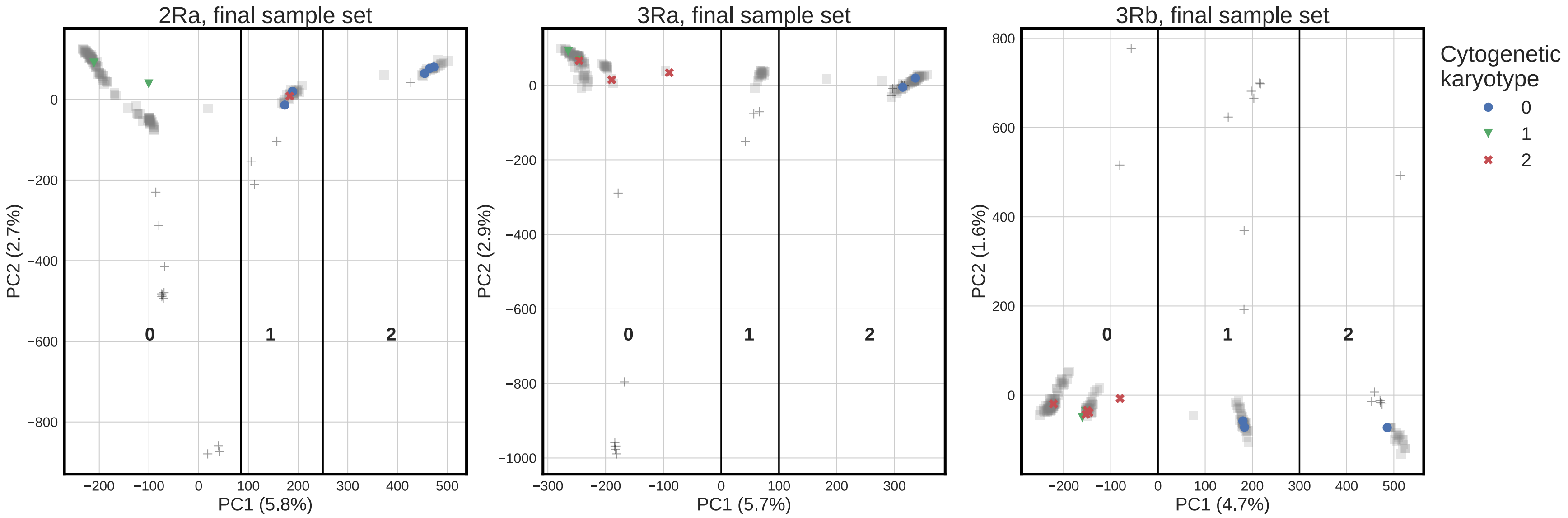

Figure S1. PCA-based genotyping of *An. funestus* inversions 2Ra, 3Ra, and 3Rb using SNPs inside the rearranged regions. Panels show PC plots obtained from the reduced population sample, as described in the main text. Black vertical lines separate the genotypes (0, 1, and 2) inferred by PCA. Circles, triangles, 'x', and squares represent specimens From Burkina Faso that were cytogenetically karyotyped. Crosses ('+') represent specimens from locations outside of Burkina Faso that were not cytogenetically karyotyped. Note that although there is some scatter along PC2, these geographically dispersed specimens still form the expected three stripes together with the specimens from Burkina Faso, along PC1. Gray squares are specimens whose cytogenetic karyotype matched the PCA-based genotype. Colored shapes are specimens whose cytogenetic karyotype (blue=0, green=1, red=2) conflicted with the PCA genotype assignment, indicative of cytogenetic misassignments.
