# Supplementary material for: High-Throughput Genotyping of Common Chromosomal Inversions in the Afrotropical Malaria Mosquito Anopheles Funestus
Source: Insects. 2020 Oct 13;11(10):693. doi: 10.3390/insects11100693 (PMC7650614; doi:10.3390/insects11100693)
Supplement: Supplementary file 1 [file insects-11-00693-s001.zip › FigS2_filtering_with_legend.pdf]

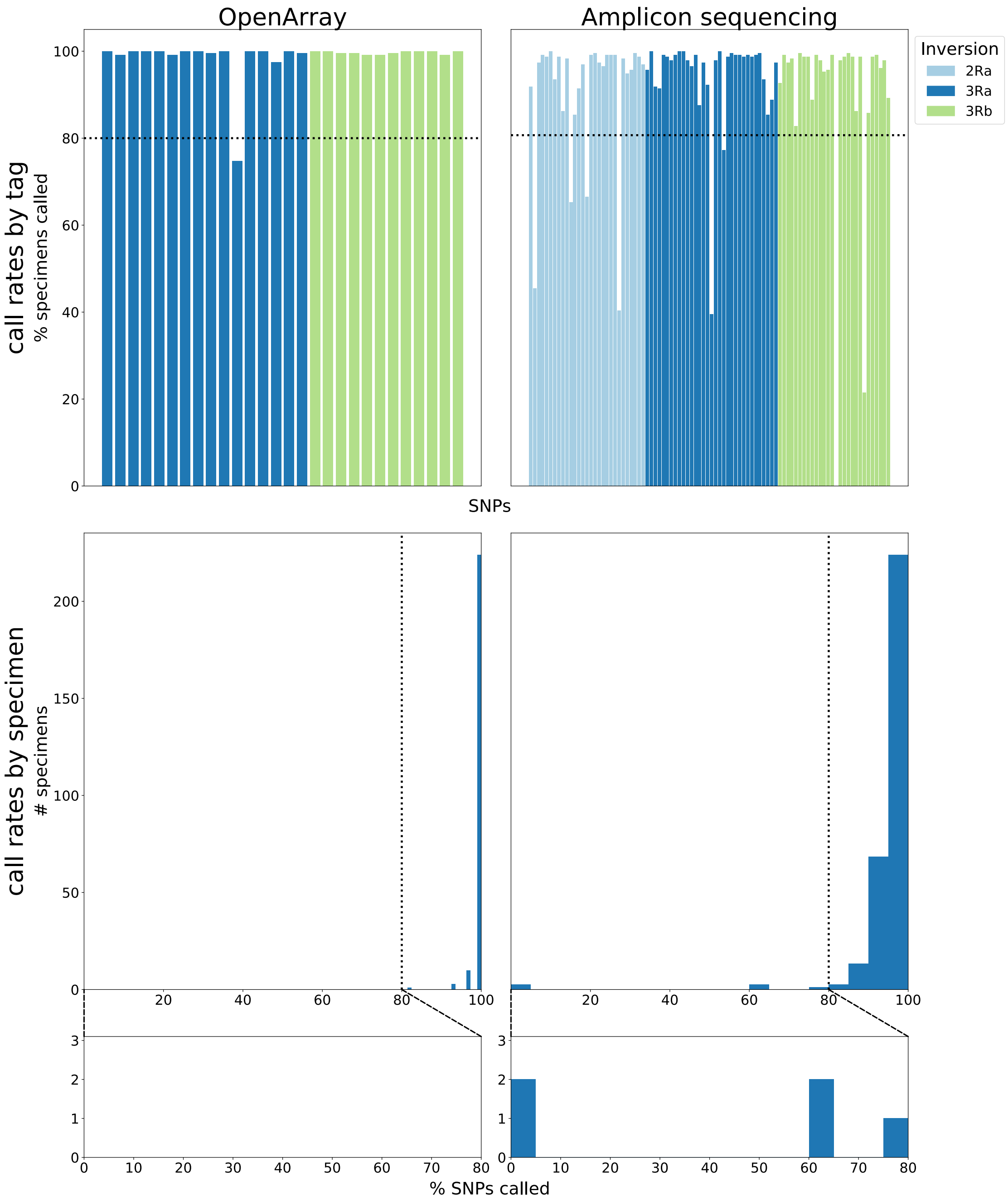

Figure S2. Tag SNP and specimen call rates for OpenArray and amplicon sequencing approaches. Bars in the top panels represent tags in the genotyping panel color-coded by inversion. Bar heights reflect the proportion of the population sample that was successfully genotyped at the focal tag. The dotted horizontal line indicates the 80% threshold below which tags were removed from the genotyping panel due to low tag call rates. The bottom panels are histograms of the number of specimens in the population panel that were successfully scored at a given proportion of tag SNPs. The vertical dotted line indicates the 80% threshold below which specimens were removed from further analysis due to low specimen call rates.
