# Supplementary figures and images for: High-Throughput Genotyping of Common Chromosomal Inversions in the Afrotropical Malaria Mosquito Anopheles Funestus
Source: Insects. 2020 Oct 13;11(10):693. doi: 10.3390/insects11100693 (PMC7650614; doi:10.3390/insects11100693)

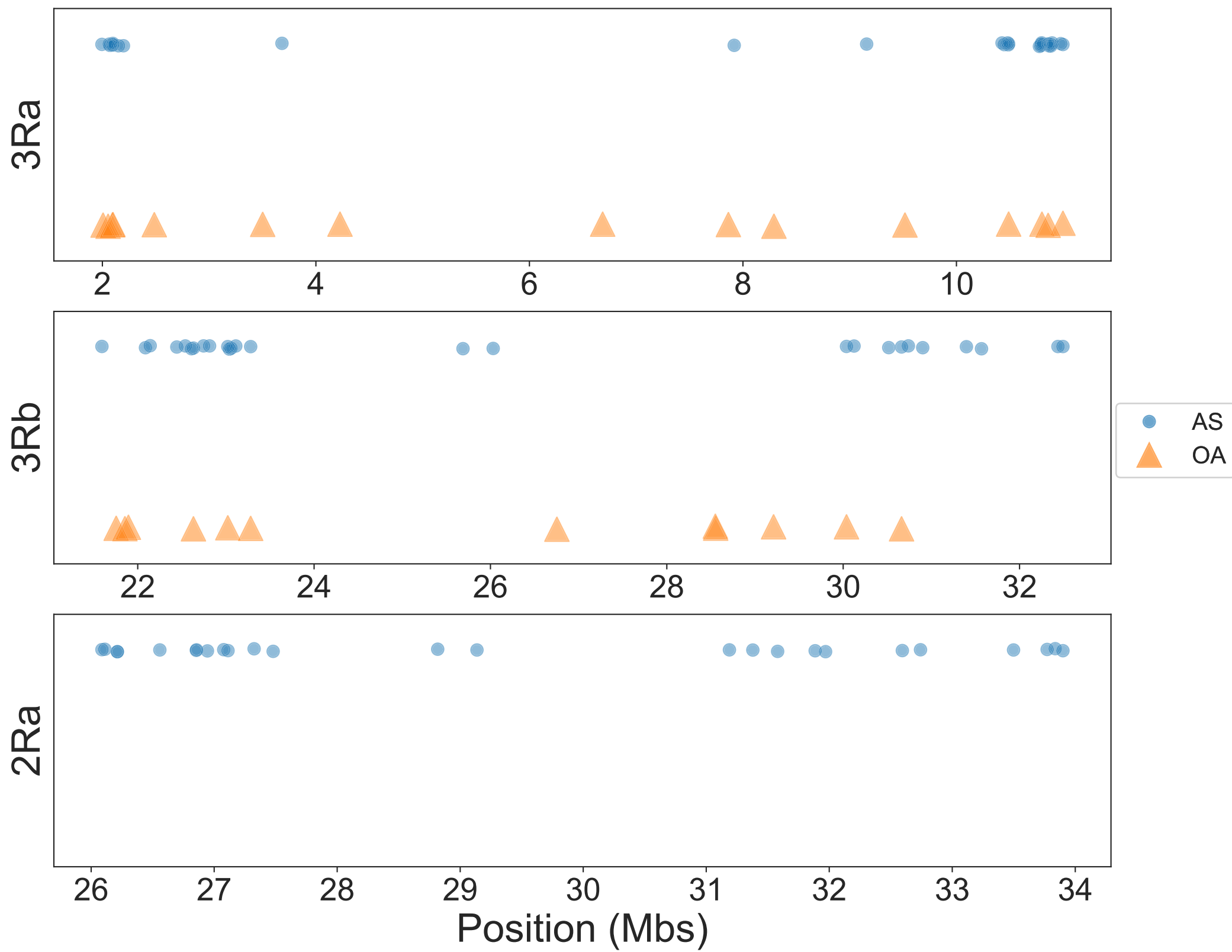

Supplement: Supplementary file 1 [file insects-11-00693-s001.zip › Fig1_tag_locations_imagefix_28Sep20.pdf]

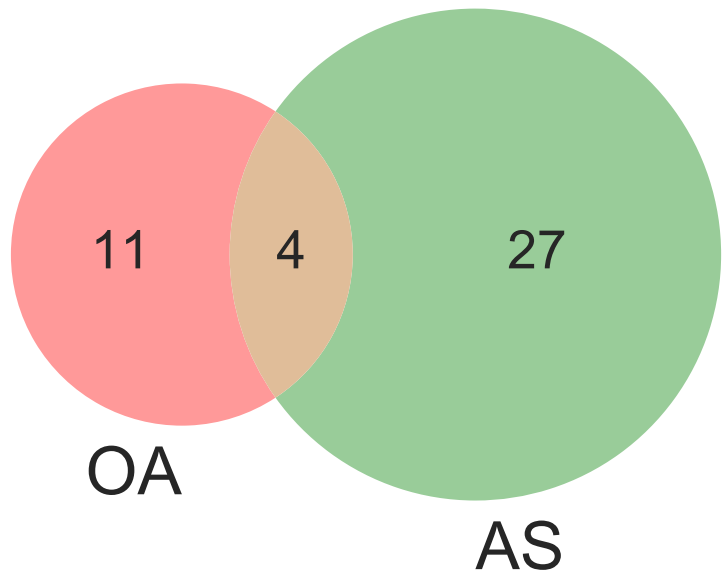

(a)

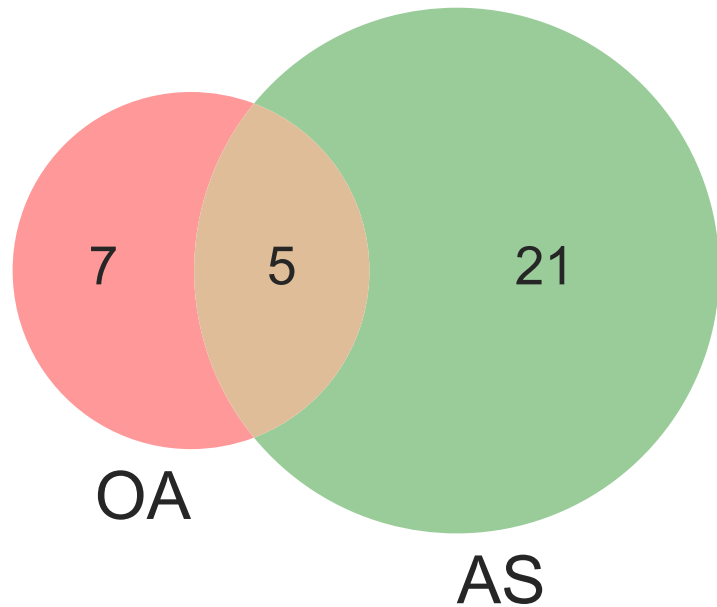

(b)

Supplement: Supplementary file 1 [file insects-11-00693-s001.zip › Fig2_tag_ven_imagefix_28Sep20.pdf]
